# Substitution of Salt with Choline Chloride in Double-Layer Flatbreads: Impact on Technological Properties and Starch Digestibility

**DOI:** 10.1007/s11130-025-01459-9

**Published:** 2026-01-26

**Authors:** Maria Santamaria, Clara Leguet, Nour Doumani, Patricia Le-Bail, Cristina M. Rosell

**Affiliations:** 1 Institute of Agrochemistry and Food Technology (IATA-CSIC), C/Agustin Escardino, 7, Paterna, Valencia, 46980 Spain; 2 Biopolymères Interactions Assemblages, INRAE, EUR 1268, BP 71627, Nantes, Cedex 3F- 44316 France; 3 Food and Human Nutritional Sciences Department, University of Manitoba, Winnipeg, Canada

**Keywords:** Bread, Gluten, gluten-free, Starch hydrolysis, Salt reduction, Breadmaking

## Abstract

The association of sodium intake with hypertension has prompted the salt reduction in food processing. However, salt reduction is technologically challenging in process like breadmaking. The aim of this study was to identify the impact of salt reduction on the dough properties, technological characteristics and *in vitro* digestion on gluten and gluten-free flatbreads. In addition, the inclusion of choline chloride, (E 1001) as salt replacer (25% choline chloride substitution) was explored. Gluten-free dough exhibited higher hardness than gluten dough due to the predominance of gelatinized starch. Salt reduction gluten-free dough presented lower hardness because of the hydrophobicity of the rice proteins. The texture of the flatbreads was not affected by the addition of choline chloride, with control and choline chloride-enriched flatbreads showing similar strength and extensibility. Moreover, gluten-free flatbreads with choline chloride presented lower starch hydrolysis, because of the ionic compound (choline chloride) on starch gelatinization, which increases the viscosity of the system. In conclusion, gluten-free flatbread with choline chloride could represent a potential strategy to decrease the glycemic index and mitigate hypertension in consumers of gluten-free bread.

**Supplementary Information:**

The online version contains supplementary material available at 10.1007/s11130-025-01459-9.

## Introduction

Excessive sodium intake is a critical concern in public health worldwide, as it is strongly associated with elevated blood pressure and an increased risk of cardiovascular diseases [1, 2]. Moreover, salt in carbohydrate-rich foods has been linked to an elevated glycemic response, because it enhances amylase activity and glucose absorption, thereby accelerating starch digestion and increasing postprandial blood glucose levels. This effect is linked to two factors: enhanced amylase activity in the presence of sodium and increased glucose uptake through sodium-dependent SGLT1 transporters [3]. Hence, the reduction of dietary sodium has emerged as a key strategy in public health nutrition.

From the technological point of view, sodium chloride promotes the development of gluten structures during dough mixing by enhancing protein-protein interactions [4, 5], modulates yeast activity during fermentation by slowing gas release, and helps regulate water activity in the final baked product [6, 7]. However, these technological effects differ in gluten-free bread, where the absence of a gluten network requires alternative structuring strategies [8]. In gluten-free formulations, sodium chloride influences starch gelatinization, water availability and hydrocolloids interactions, affecting dough viscosity and stability, and crumb texture [9, 10]. Although reducing salt intake in bread would be nutritionally beneficial, it presents significant technological challenges, as salt plays a crucial role in the technological properties of bread, such as, fermentation rate, dough rheology and texture, as well as its organoleptic attributes [11, 12]. Although reducing salt in bread offers clear nutritional benefits, it poses major technological challenges because salt strongly influences fermentation rate, dough rheology, texture, and overall sensory quality [11, 12]. While reported work has clarified salt’s role in gluten network formation and bread structure [12], it provides only a partial view of its technological function. Research to date has largely overlooked how salt reduction affects non-gluten systems, alternative formulations, and processes where bread structure is not dominated by gluten aeration. These limitations highlight the need for broader investigations that extend beyond traditional gluten-based bread models.

To address these challenges, one promising strategy is the partial replacement of salt with choline chloride (CC), a compound recognized for its functional properties in food systems and classified as an emulsifier additive (E 1001) in the Codex Alimentarius [11, 13]. Choline chloride impact on the breadmaking process has been ascribed to the increased viscosity of starch after gelatinization due to the reduction of the available water fraction [14]. Le-Bail et al. [11] reported at the CIGR Section VII Technical Symposium (Guangzhou, China, 2013) that the substitution of 25% of salt with choline chloride in pizza dough had no negative impact on flavor or perceived saltiness. Similarly, Crucean et al. [11] optimized a reduced-salt bread recipe (50% of salt) enriched with CC (25, 45, 65, 85%) content, and evaluated its sensory acceptability among French consumers, revealing that CC enhanced the perception of saltiness in reduced-salt bread. Nonetheless, no single formulation was preferred across consumer subgroups, as acceptance depended heavily on individual eating habits and perceptions of food enrichment. Additional benefits of CC are its softening effect observed in bread fortified with 25% CC, which was associated with its impact on starch gelatinization and retrogradation [4].

Despite the interest in reducing the salt content in bakery products, this reduction has been only explored in fermented aerated breads, with limited research in other type of breads. Georges et al. [5] investigated the salt reduction in white pita bread using three types of salt (NaCl, Ag-NaCl, and NaCl-KCl) using five different levels (0.3, 0.6, 0.9, 1.2, and 1.5%), showing that salt-reduced pita breads could be attainable with 0.6% salt having no major difference in texture.

Considering the limited existing information about salt-reduced flatbreads, gluten and gluten-free, additional research might contribute to improve their healthiness while enhancing its technological properties. Choline chloride might be an innovative alternative for salt reduction in double-layer flatbreads, with a concomitant impact on bread features and health-related aspects such as the glycemic index. Therefore, the objective of this study was to develop gluten and gluten-free double-layer flatbread recipes with a 50% reduction in salt, partially replaced (25%) by choline chloride. The impact of these modifications was analyzed in terms of dough properties, technological parameters (baking loss, moisture content, texture, color), and in vitro starch digestibility using an enzymatic method.

## Supplementary Methods

Detailed procedures for breadmaking process, dough characteristics, flatbread technological properties 16, starch *in vitro* digestion, and statistical analysis used are provided in the supplementary materials.

## Results and Discussion

### Dough Properties

Dough properties are summarized in Table 1. As expected, flatbreads made with wheat flour exhibited higher expansion during proofing compared to those made of gluten-free recipes. Gluten free-doughs, even containing hydrocolloids, have lower ability than wheat doughs to hold the carbon dioxide produced during the fermentation. Rosell et al. [17] reported that the absence of gluten in rice breads limits the fermentation process due to the reduced capacity of gluten-free proteins to retain the carbon dioxide released during proofing. As it was expected the hardness of the GF dough was higher than the one observed with the wheat dough. It is well-known that gluten-free doughs are harder than the ones made with wheat [18] and it has been a great concern in flatbread [19, 20]. The reduction in salt content of the gluten and gluten-free doughs had no statistically significant impact on proofing volume and moisture content. However, the dough texture presented statistically significant differences (*p* < 0.05) in both types of doughs, although opposite effects were observed. Specifically in gluten containing dough, the reduction of salt provoked an increase of the dough hardness, likely due to the changes in the proteins’ interactions, but that hardness increase was ameliorated with the presence of CC. The interaction of sodium chloride with water molecules and polypeptides facilitates the formation of hydrogen bonds among gluten protein chains [12]. Wang et al. [21] showed that the use of low-sodium salts enhanced gluten strength in wheat dough, an effect attributed to the capacity of Na⁺, K⁺ and Cl⁻ ions to screen electrostatic charges on gluten proteins. Nevertheless, the effect was not observed in the gluten-free doughs, likely due to the higher hydrophobicity of the rice proteins, compared to wheat gluten. Rice proteins show low solubility due to their high hydrophobic amino acid content and extensive intermolecular bonding [22]. In gluten-free systems, their high surface hydrophobicity has been shown to limit water binding and potentially slow starch retrogradation [23]. Consequently, reducing salt content results in a softer dough. This opposite response can be explained by the fact that in gluten-free systems the mechanical properties are dominated by starch. Sodium chloride can compete with starch granules for water molecules, lowering water mobility and delaying gelatinization, which in turn limits starch swelling and weakens the viscoelastic matrix [14].

**Table 1:** Impact of salt reduction and choline chloride on the moisture and dough characteristics of gluten and gluten-free doughs

Samples	Proofing volume (mL/100 g)	Dough moisture (%)	Dough hardness (g)
**Gluten**			
G-C	43 ± 10	47.4 ± 0.2	827 ± 33^b^
G-Red	50 ± 0	47.2 ± 0.5	1008 ± 123^a^
G-CC	48 ± 3	47.7 ± 0.5	925 ± 67^ab^
*p-value*	*0.5630*	*0.3361*	***0.0067***
**Gluten-free**			
GF-C	27 ± 3	45.4 ± 0.1	3911 ± 140^a^
GF-Red	29 ± 0	45.7 ± 0.2	3473 ± 298^b^
**GF-CC**	29 ± 0	45.5 ± 0.1	3467 ± 377^b^
*p-value*	*0.4648*	*0.1757*	***0.0256***

*Means with different letters within a column and type of bread indicate significant differences (*p *< 0.05). Meaning of the samples’ codes: Gluten (G) or gluten-free (GF) doughs with common amount of salt (C), 50% reduced salt content (Red), and 50% reduced salt content added with 25% choline chloride (CC)

## Technological Properties of Flatbreads

Recipes were defined to reduce the salt content in FB. The theoretical salt content in the resulting FB was 0.7 g/100 g FB, compared to the 1.2 g/100 g of salt in the control FB. Technological properties of the double-layer flatbread exhibited statistically significant differences (*p* < 0.05), except for FB moisture and extensibility in G-FB; and extensibility in GF-FB (Table 2). In the gluten containing FB, salt reduction resulted in lower baking loss, which directly result in higher moisture content (G-Red). However, the CC contributed to reduce the baking loss, although it did not result in a significant change in the moisture content (G-CC).

The control gluten-free FB (GF-C) showed reduced baking loss, with higher retention of water. Similar findings in gluten-free flatbread were reported by Mohammadi et al. [24]. However, formulations with xanthan gum and carboxymethyl cellulose exhibited even higher moisture content, which was attributed to their high water-binding capacity. In contrast to the observations in gluten containing FB, gluten-free FB with reduced salt (GF-Red) exhibited higher baking loss and the subsequent reduction of the FB moisture content. As previously discussed, the presence of salt in the gluten dough contributes to the formation of hydrogen bonds and electrostatic interactions with protein molecules [12]. For this reason, G-Red still provides a structural barrier that limits water evaporation, resulting in lower baking loss and relatively higher final moisture. In contrast, in gluten-free systems (GF-Red), sodium chloride can compete with starch granules for water, reducing water mobility and delaying gelatinization [14]. This limits granule swelling and weakens the viscoelastic matrix, leading to increased water loss during baking and lower moisture content. The presence of CC reduced the baking loss of the gluten-free FB, but it did not impact on the resulting moisture content.

Regarding texture parameters (Table 2), strength presented statistically significant differences (*p* < 0.05) in both sample groups. Salt reduction decreased the strength of the gluten FB, but without impacting in the gluten-free FB. But in the case of the extensibility, gluten and gluten-free FB with reduced salt content showed significantly higher extensibility. As mentioned above, in gluten FB, a lower salt content may reduce protein interactions and increase the moisture in the flatbread, which could promote greater extensibility. However, in gluten-free FB, which depend primarily on starch for structure, reduced salt enhances water availability for starch hydration and partial gelatinization, increasing the extensibility of the starch-based matrix. The addition of CC resulted in strength and extensibility values comparable to those of the control recipe. This highlights the optimal performance of choline chloride as a salt substitute and its functional role in dough properties.

**Table 2:** Effect of salt reduction on the technological properties of gluten and gluten-free double-layer flatbreads

Samples	Baking loss (%)	Moisture (%)	Tensile texture analysis	
			Strength (*N*)	Extensibility (mm)
**Gluten FB**				
G-C	27.4 ± 0.3^a^	28.1 ± 0.9^b^	5.7 ± 1.1^a^	12.9 ± 4.7^b^
G-Red	21.4 ± 1.0^c^	32.1 ± 1.5^a^	3.7 ± 1.0^b^	16.6 ± 2.5^a^
G-CC	23.1 ± 0.6^b^	29.6 ± 1.1^ab^	5.0 ± 0.6^a^	11.7 ± 4.0^b^
*p-value*	***0.0001***	*0.0967*	***0.0012***	*0.0504*
**Gluten-free FB**				
GF-C	21.0 ± 1.1^c^	29.7 ± 1.6^a^	7.9 ± 0.6^ab^	4.4 ± 1.2^a^
GF-Red	28.3 ± 1.5^a^	26.3 ± 1.5^b^	8.8 ± 1.9^a^	5.6 ± 1.2^b^
GF-CC	25.6 ± 0.6^b^	26.2 ± 1.2^b^	6.4 ± 0.2^b^	4.7 ± 0.5^ab^
*p-value*	***0.0000***	***0.0316***	***0.0123***	*0.0692*

*Means with different letters within a column and type of bread were significantly different (*p* < 0.05). Meaning of the samples’ codes: Gluten (G) or gluten-free (GF) doughs with common amount of salt (C), 50% reduced salt content (Red), and 50% reduced salt content added with 25% choline chloride (CC)

**Fig. 1:**
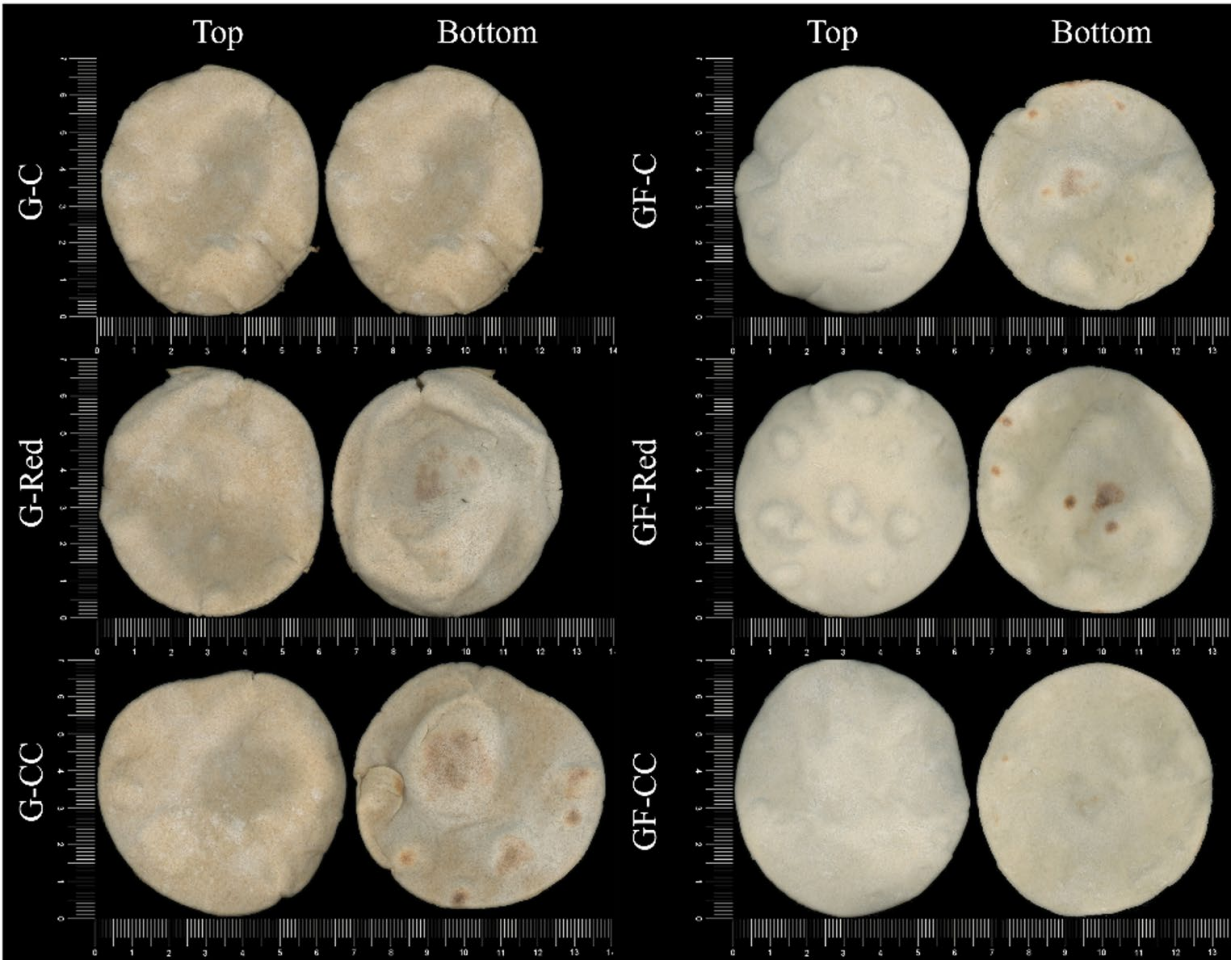
Top and bottom side of gluten and gluten-free double-layer flatbreads with different amount of salt. Meaning of the samples’ codes: Gluten (G) or gluten-free (GF) doughs with common amount of salt (C), 50% reduced salt content (Red), and 50% reduced salt content added with 25% choline chloride (CC)

As shown in Fig. 1, the gluten-containing samples exhibited a brownish hue, whereas the gluten-free FB displayed a predominantly whitish color, which can be attributed to initial flour difference. Consequently, the gluten FB showed higher reddish tones (+ a*), while gluten-free FB exhibited greater luminosity (L*) (Table 3). This lighter appearance in the gluten-free FB is also consistent with its higher starch and lower protein content, which limits the extent of Maillard reaction and caramelization during baking. Additionally, slight brownish hues were observed on the bottom surfaces of the flatbreads, due to baking in a deck oven.

**Table 3:** Color determination of gluten and gluten-free double-layer flatbreads (top, burnt bottom, and bottom sides)

Sample	Top side			Burnt bottom side			Bottom side		
	L*	a*	b*	L*	a*	b*	L*	a*	b*
**Gluten FB**									
G-C	74.30 ± 1.94	2.31 ± 0.37	18.01 ± 0.94	64.44 ± 3.95	5.93 ± 1.92^a^	20.82 ± 3.50^b^	76.17 ± 1.25	1.27 ± 0.72^b^	11.85 ± 1.14^b^
G-Red	75.37 ± 1.84	2.13 ± 0.51	17.28 ± 1.02	70.43 ± 1.97	2.57 ± 0.38^b^	28.83 ± 2.87^a^	74.28 ± 2.59	2.57 ± 0.75^a^	18.58 ± 1.23^a^
G-CC	74.25 ± 1.63	2.37 ± 0.39	18.72 ± 0.79	69.02 ± 3.57	4.88 ± 1.61^ab^	20.15 ± 1.63^b^	75.18 ± 1.19	2.12 ± 0.28^a^	17.86 ± 1.61^a^
*p-value*	*0.5630*	*0.6200*	*0.1469*	*0.1335*	*0.0511*	***0.0067***	*0.3937*	***0.0122***	***0.0000***
**Gluten-free FB**									
GF-C	82.25 ± 0.65^b^	0.38 ± 0.10	15.01 ± 1.21^a^	67.02 ± 2.64^c^	8.64 ± 1.44^a^	27.04 ± 1.65^a^	81.98 ± 0.60^b^	0.17 ± 0.03	15.83 ± 1.10^a^
GF-Red	83.46 ± 0.78^a^	0.39 ± 0.04	13.12 ± 0.38^b^	76.62 ± 3.57^b^	1.72 ± 0.55^b^	16.14 ± 1.70^b^	83.12 ± 1.13^a^	0.44 ± 0.20	13.30 ± 1.71^b^
GF-CC	83.14 ± 0.69^a^	0.42 ± 0.02	13.85 ± 1.04^ab^	81.70 ± 1.35^a^	0.98 ± 0.06^b^	15.54 ± 1.25^b^	83.04 ± 0.53^a^	0.36 ± 0.08	14.51 ± 1.16^ab^
*p-value*	***0.0071***	*0.8888*	***0.0132***	***0.0000***	***0.0000***	***0.0000***	***0.0118***	*0.1011*	***0.0045***

*Means with different letters within a column were significantly different (p < 0.05). Meaning of the samples’ codes: Gluten (G) or gluten-free (GF) doughs with common amount of salt (C), 50% reduced salt content (Red), and 50% reduced salt content added with 25% choline chloride (CC)

Salt reduction impacted more on the color of the gluten-free FB, being significant the increase in the luminosity and the reduction of the brownish tone (+ b*) in the different sides of the breads. Moreover, in the burnt bottom side salt reduction led to lower reddish tones (+ a*). Conversely, the salt reduction in the gluten FB only significantly increased the a* and b* values of the bottom side. The addition of CC had only a minor effect on the color of the reduced salt FB. The G-CC FB did not show significant differences with the reduced salt sample (G-Red), except for the reduction in b* of the burnt bottom side. Nevertheless, in gluten-free FB, GF-CC showed higher luminosity in the burnt bottom side than the GF-Red. Therefore, salt reduction impacted FB color, but the extend was dependent on the type of flour used, being more prominent on the GF FB made with rice flour.

### Flatbread *InVitro* Digestion

**Fig. 2:**
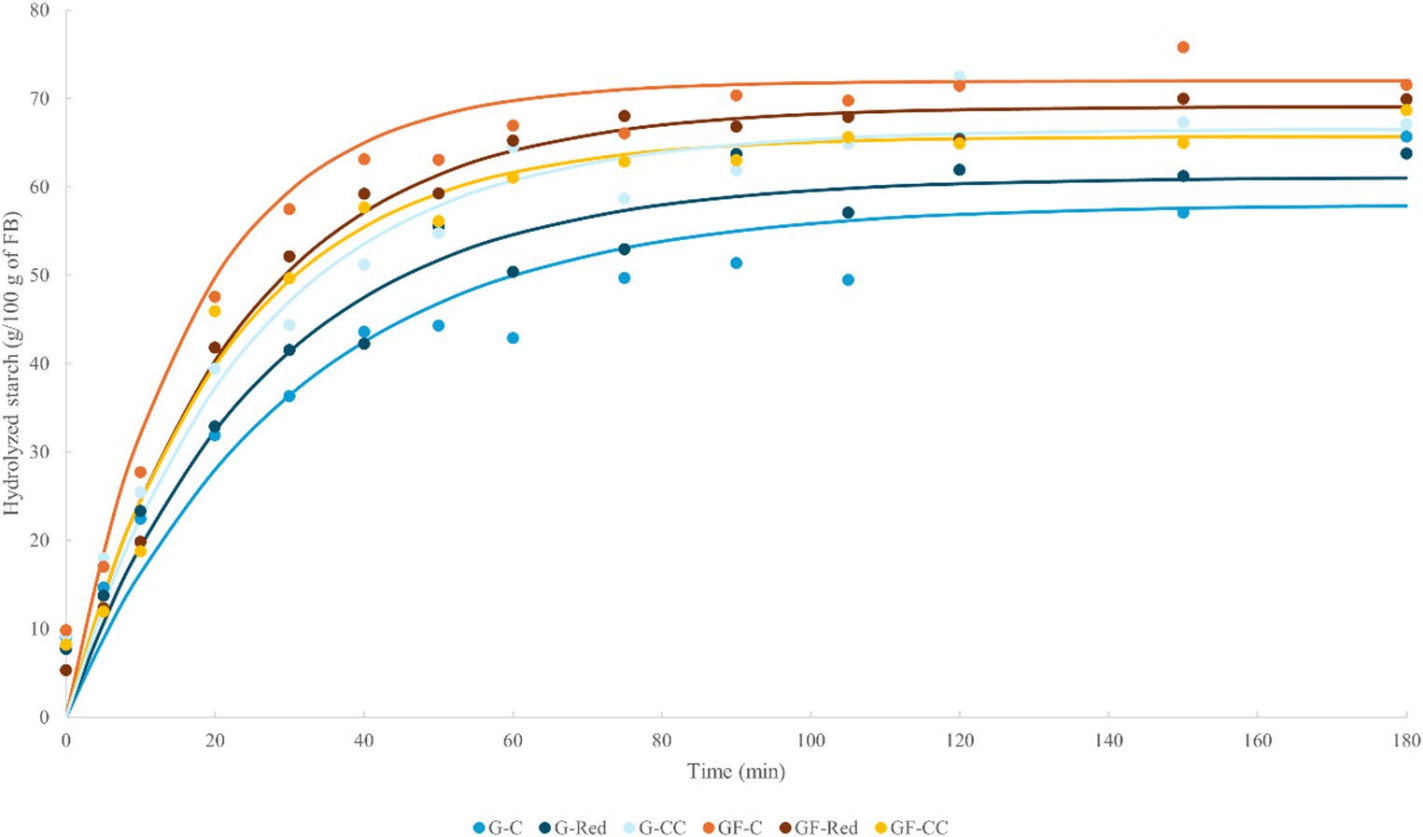
Starch *in vitro* digestion of gluten and gluten-free double-layer flatbreads. Experimental (•) and modelled (−) data. Meaning of the samples’ codes: Gluten (G) or gluten-free (GF) doughs with common amount of salt (C), 50% reduced salt content (Red), and 50% reduced salt content added with 25% choline chloride (CC).

The flatbread in vitro hydrolysis plots are displayed in Fig. 2. It was observed that the gluten-free FB samples showed higher starch hydrolysis compared to the gluten-containing FB. This is associated with the high glycemic index of gluten-free products, as it has been reported in different articles [18, 25]. The starch hydrolysis parameters are detailed in Table 4. Salt reduction in the FB did not produce significant impact on the starch hydrolysis of the gluten and gluten-free FB, except for the RS. In gluten FB, salt reduced samples (G-Red; G-CC) presented higher starch hydrolysis than control sample (G-C), as indicated the kinetic parameters, although it was only significant in the presence of CC. Conversely, Thorburn et al. [3] established a positive correlation between higher starch hydrolysis and the salt intake in carbohydrate rich foods, possibly due to the modulatory effect of salt on amylase activity or intestinal glucose absorption. The present study observed this trend in gluten-free FB, where the starch hydrolysis decreased with salt reduced formulations, but hydrolysis parameters did not show statistically significant differences. GF-Red and GF-CC presented lower rapidly digestible starch (RDS), and higher slowly digestible starch (SDS) and resistant starch (RS) than gluten-free control flatbread (GF-C). These results could be associated with a lower estimated glycemic response, since RDS (digested within the first 20 min) is the fraction primarily responsible for the rapid postprandial increase in blood glucose. In contrast, the higher proportions of SDS (digested within 20–120 min) and RS (not digested in the small intestine) indicate a slower starch hydrolysis rate, which is linked to lower glycemic index values [15]. Furthermore, the modelled data using the nonlinear Exponential model of Box-Lucas **Eq. (1)** (see in SI) showed a decrease in the hydrolysis rate (*k*), maximum concentration (C_∞_), and area under the curve for salt-reduced GF flatbreads. Crucean et al. [14] indicate that CC is an ionic compound like NaCl, which increases the viscosity of the aqueous solution, generating an impact on starch gelatinization. In fact, connected with this, our previous study correlates negatively the gels viscosity with the starch hydrolysis, affecting the alpha-amylase activity [15]. Again, the trend observed due to salt reduction was completely dependent on the type of flour used for breadmaking.

**Table 4:** Starch hydrolysis parameters of double-layer gluten and gluten-free flatbreads

Samples	RDS (g/100 g)	SDS (g/100 g)	DS (g/100 g)	RS (g/100 g)	C∞	k (min^− 1^)	AUC
**Gluten FB**							
G-C	27.99 ± 3.36	28.61 ± 0.39	69.57 ± 1.06	15.91 ± 0.37^a^	56.64 ± 1.94^b^	0.032 ± 0.001	8,665 ± 709
G-Red	32.40 ± 6.25	27.96 ± 1.27	60.72 ± 5.06	13.27 ± 0.13^b^	61.11 ± 4.47^ab^	0.038 ± 0.007	9,350 ± 974
G-CC	37.25 ± 3.24	28.77 ± 4.21	68.80 ± 4.65	14.34 ± 0.92^ab^	66.57 ± 1.39^a^	0.041 ± 0.007	10,326 ± 54
*p-value*	*0.2670*	*0.9469*	*0.1934*	***0.0441***	*0.0933*	*0.3728*	*0.2004*
**Gluten-free FB**							
GF-C	49.72 ± 5.16	22.21 ± 3.74	75.45 ± 1.05	16.23 ± 1.30^b^	72.01 ± 1.35^a^	0.059 ± 0.010	11,702 ± 417^a^
GF-Red	40.29 ± 1.60	28.40 ± 0.36	63.95 ± 7.38	18.97 ± 0.42^a^	69.05 ± 1.18^ab^	0.044 ± 0.002	10,831 ± 241^ab^
GF-CC	39.76 ± 3.13	25.66 ± 1.05	72.98 ± 1.99	17.52 ± 0.01^ab^	65.68 ± 1.97^b^	0.047 ± 0.004	10,387 ± 425^b^
*p-value*	*0.1154*	*0.1512*	*0.1551*	*0.0883*	*0.0582*	*0.1562*	*0.0811*

*Means within the same column followed by different letters indicate significant differences (*p* < 0.05). Meaning of the samples’ codes: Gluten (G) or gluten-free (GF) doughs with common amount of salt (C), 50% reduced salt content (Red), and 50% reduced salt content added with 25% choline chloride (CC)

## Overall Features of Double-Layer Flatbreads

**Fig. 3:**
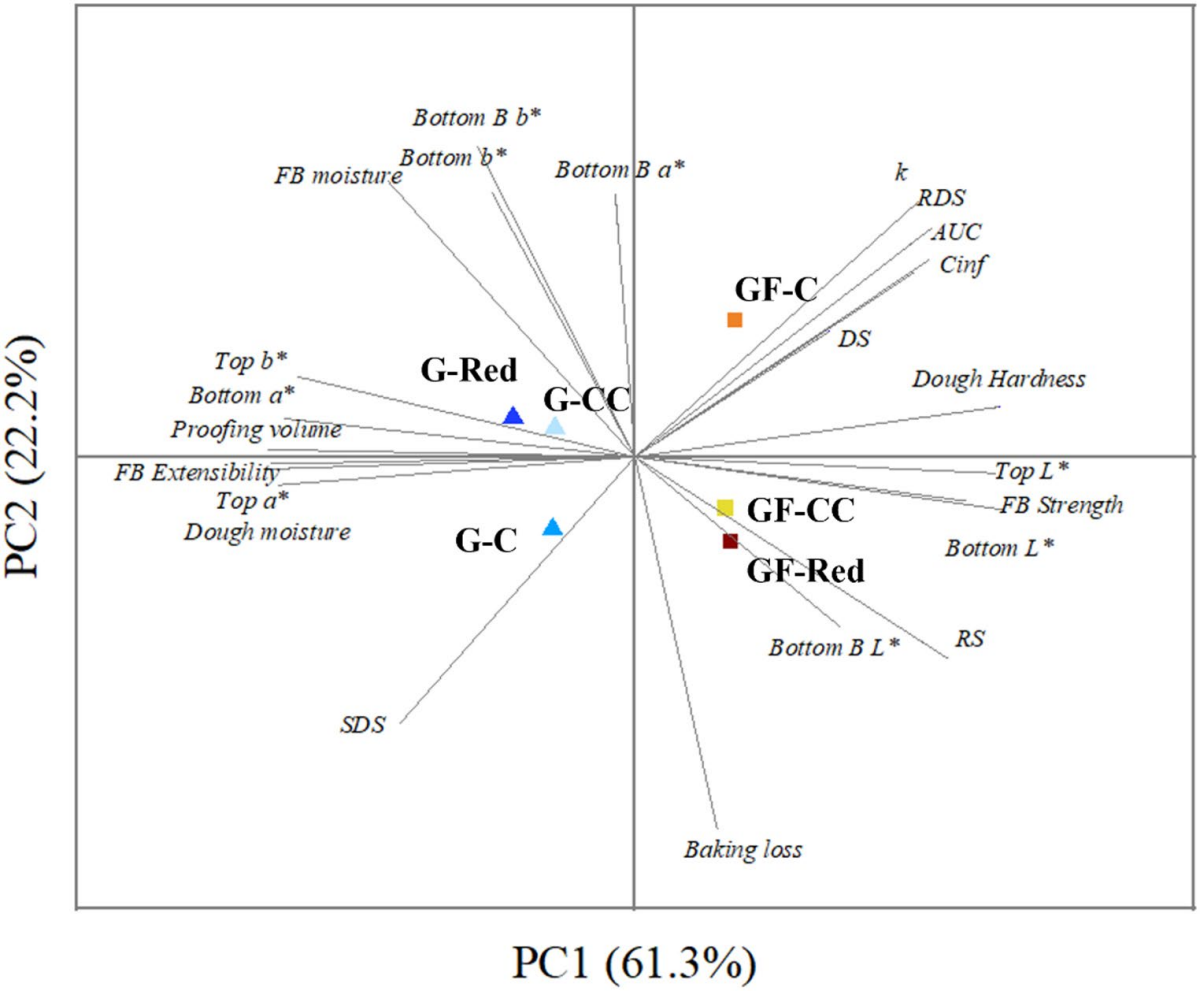
Principal Component Analysis (PCA) plot comparing technological and digestion parameters from gluten and gluten-free salt reduced double-layer flatbreads

A principal component analysis (PCA) (Fig. 3) was conducted to discern potential clusters based on salt-reduced gluten and gluten-free flatbreads recipes, using technological properties and digestion parameters as studied factors. This analysis accounts for 84% of the variance observed among the recipes. Principal component 1 (PC1) accounted for 61.3% of the variability, while principal component 2 (PC2) explained 22.2%. PC1 is primarily influenced by dough hardness and kinetic parameters of starch hydrolysis (*k*, C_∞_, AUC, RDS), while proofing volume, dough moisture and extensibility, and the a* value have an opposite influence. In PC2, flatbread moisture, b* and a* values represent the driving variables while an opposite effect was observed for RS, and baking loss. Figure 3 clearly shows that PC1 differentiates gluten-free flatbreads and PC2 distinguishes gluten flatbreads, both separating salt-reduction recipes from control recipes, confirming the impact of salt reduction on technological and digestion parameters. The PCA validates the results mentioned above. Gluten dough presented higher proofing volume and moisture content, but gluten-free dough exhibited higher hardness. These textural variations are also reflected in the flatbread texture, where gluten FB displayed higher strength, while gluten-free FB exhibited greater extensibility. Regarding color, gluten flatbreads showed more reddish tones corresponding to a* values, while gluten-free FB showed greater luminosity (L*), likely due to differences in flour composition. Additionally, the resistant starch (RS) content had a greater impact on gluten-free samples with reduced salt content, influencing digestion-related parameters. The PCA also revealed that salt reduction FB, in the absence or presence of CC revealed completely opposite behavior in the gluten or gluten free FB. Particularly due to the impact on RS, while in the case of GF FB the reduction of salt promoted an increase in the RS, the opposite behavior was observed in the case of G-FB. The impact of salt reduction in gluten FB seems to be governed by the influence of salt reduction on proteins interaction, while in the case of GF FB the effect is governed by the interaction with starch.

## Conclusions

The reduction of 50% salt content in the recipes of double-layer flatbreads led to significant differences in technological properties and digestion parameters. The addition of choline chloride could mitigate those differences, resembling the performance of its salt containing counterpart. In gluten flatbreads, choline chloride primarily improves the technological properties. Its incorporation (G-CC) maintained moisture content and texture parameters like the control flatbread (G-C). In addition, gluten flatbreads exhibited a brownish hue, and salt reduction resulted in a burnt bottom side, which could affect the quality of the final product. However, gluten flatbread with choline chloride (G-CC) increased starch hydrolysis compared to the gluten control (G-C). In the other hand, the suggested gluten-free recipe (GF-CC) could be an alternative formulation to reduce the glycemic index and mitigate hypertension based on its performance results. Choline chloride incorporation in gluten-free flatbreads (GF-CC) resulted in lower and slower starch hydrolysis compared to the control flatbread (GF-C), making it a promising alternative for formulating gluten-free breads with a reduced impact on glycemic response and sodium intake.

## Supplementary Information

Below is the link to the electronic supplementary material.

Supplementary Material 1 (DOCX 20.0 KB)

## Data Availability

Authors will make data available upon request.

## Abbreviations

ANOVA: Analysis of variance
AUC: Area under the curve
C: Control
CC: Choline chloride
DS: Digestible starch
FB: Flatbread
G: Gluten
GF: Gluten-free
GOPOD: Glucose oxidase–peroxidase
ISO: International Organization for Standardization
PCA: Principal Component Analysis
RDS: Rapidly digestible starch
Red: Salt-reduced
RS: Resistant starch
SDS: Slowly digestible starch

## References

[1] WHO (2021) WHO global sodium benchmarks for different food categories

[2] Weegels PL (2019) The future of bread in view of its contribution to nutrient intake as a starchy staple food. Plant Foods Hum Nutr 74:1–9. 10.1007/s11130-019-0713-6 30637605 10.1007/s11130-019-0713-6

[3] Thorburn AW, Brand JC, Truswell AS (1986) Salt and the glycaemic response. BMJ 292:1697–1699. 10.1136/bmj.292.6537.1697 3089360 10.1136/bmj.292.6537.1697 PMC1340630

[4] Crucean D, Pontoire B, Debucquet G, Le-Bail A, Le-Bail P (2023) The use of choline chloride for salt reduction and texture enhancement in bread. Appl Food Res 3:100371. 10.1016/j.afres.2023.100371

[5] Georges C, Daroub H, Toufeili I, Isma’eel H, Olabi A (2018) Dough mixing properties and white pita bread sensory characteristics as affected by salt reduction. Int J Food Prop 21:2578–2589. 10.1080/10942912.2018.1540987

[6] Belz MCE, Ryan LAM, Arendt EK (2012) The impact of salt reduction in bread: a review. Crit Rev Food Sci Nutr 52:514–524. 10.1080/10408398.2010.502265 22452731 10.1080/10408398.2010.502265

[7] Van Rooyen J, Simsek S, Oyeyinka SA, Manley M (2023) Wheat starch structure–function relationship in breadmaking: a review. Compr Rev Food Sci Food Saf 22:2292–2309. 10.1111/1541-4337.13147 37010110 10.1111/1541-4337.13147

[8] Matos Segura ME, Rosell CM (2011) Chemical composition and starch digestibility of different gluten-free breads. Plant Foods Hum Nutr 66:224. 10.1007/s11130-011-0244-2 21769691 10.1007/s11130-011-0244-2

[9] Arendt EK, Dal Bello F (2008) Gluten-free breads. Gluten-free cereal products and beverages. Academic, San Diego, pp 289–307

[10] Li C (2022) Recent progress in understanding starch gelatinization - an important property determining food quality. Carbohydr Polym 293:119735. 10.1016/j.carbpol.2022.119735 35798430 10.1016/j.carbpol.2022.119735

[11] Crucean D, Debucquet G, Rannou C, Le-Bail A, Le-Bail P (2018) Vitamin B4 as a salt substitute in bread: a challenging and successful new strategy. Sensory perception and acceptability by French consumers. Appetite 134:17–25. 10.1016/j.appet.2018.12.020 30576725 10.1016/j.appet.2018.12.020

[12] Hoeller N, Scherf KA (2024) Influence of salts on the protein composition and functionality of gluten. J Cereal Sci 118:103978. 10.1016/j.jcs.2024.103978

[13] Pashaei M, Zare L, Sadrabad EK, Abad AHS, Mollakhalili-Meybodi N, Abedi A-S (2021) The impacts of salt reduction strategies on technological characteristics of wheat bread: a review. J Food Sci Technol 59:4141–4151. 10.1007/s13197-021-05263-6 36193481 10.1007/s13197-021-05263-6 PMC9525553

[14] Crucean D, Pontoire B, Debucquet G, Le-Bail A, Le-Bail P (2021) Influence of the presence of choline chloride on the classical mechanism of gelatinization of starch. Polymers 13:1509. 10.3390/polym13091509 34067213 10.3390/polym13091509 PMC8125809

[15] Santamaria M, Garzon R, Moreira R, Rosell CM (2021) Estimation of viscosity and hydrolysis kinetics of corn starch gels based on microstructural features using a simplified model. Carbohydr Polym 273:118549. 10.1016/j.carbpol.2021.118549 34560961 10.1016/j.carbpol.2021.118549

[16] Gasparre N, Garzon R, Marín K, Rosell CM (2024) Exploring the integration of orange peel for sustainable gluten-free flatbread making. LWT 198:115969. 10.1016/j.lwt.2024.115969

[17] Rosell CM, Benavent-Gil Y, Garzon R (2021) Rice flour breads. <book-title update="added">Trends in Wheat and Bread Making. Academic Press, London, pp 405–429

[18] Matos ME, Rosell CM (2015) Understanding gluten-free dough for reaching breads with physical quality and nutritional balance. J Sci Food Agric 95:653–661. 10.1002/jsfa.6732 24816770 10.1002/jsfa.6732

[19] Santamaria M, Ruiz M, Garzon R, Rosell CM (2024) Comparison of vegetable powders as ingredients of flatbreads: technological and nutritional properties. Int J Food Sci Technol 59:7203–7212. 10.1111/ijfs.17441

[20] Santamaria M, Garzon R, Skendi A, Papageorgiou M, Rosell CM (2025) Examining the role of polyphenols in the in vitro starch hydrolysis of gluten free enriched flatbreads. Food Chem 486:144456. 10.1016/j.foodchem.2025.144456 40334487 10.1016/j.foodchem.2025.144456

[21] Wang X, Liang Y, Wang Q, Zhang X, Wang J (2021) Effect of low-sodium salt on the physicochemical and rheological properties of wheat flour doughs and their respective gluten. J Cereal Sci 102:103371. 10.1016/j.jcs.2021.103371

[22] Yang J, Meng D, Wu Z, Chen J, Xue L (2023) Modification and solubility enhancement of rice protein and its application in food processing: a review. Molecules 28(10):4078. 10.3390/molecules28104078 37241820 10.3390/molecules28104078 PMC10223372

[23] Waziiroh E, Bender D, Fuhrmann PL, Schoenlechner R (2024) Effect of non-covalent interactions on gluten-free batter stability and bread properties. LWT 215:117263. 10.1016/j.lwt.2024.117263

[24] Mohammadi M, Sadeghnia N, Azizi M-H, Neyestani T-R, Mortazavian AM (2013) Development of gluten-free flat bread using hydrocolloids: xanthan and CMC. J Ind Eng Chem 20:1812–1818. 10.1016/j.jiec.2013.08.035

[25] Capriles VD, Arêas JAG (2016) Approaches to reduce the glycemic response of gluten-free products: in vivo and in vitro studies. Food Funct 7:1266–1272. 10.1039/C5FO01264C 26838096 10.1039/c5fo01264c

