# Supplementary material for: Substitution of Salt with Choline Chloride in Double-Layer Flatbreads: Impact on Technological Properties and Starch Digestibility
Source: Plant Foods Hum Nutr. 2026 Jan 26;81(1):16. doi: 10.1007/s11130-025-01459-9 (PMC12835044; doi:10.1007/s11130-025-01459-9)
Supplement: Supplementary file 1 — Supplementary Material 1 (DOCX 20.0 KB) [file 11130_2025_1459_MOESM1_ESM.docx]

**MATERIALS AND METHODS**

**Materials**

Flours used in this study included: a gluten containing T80 wheat flour acquired from Meunerie Guenego (Saint Germain du corbeis, France); and a gluten-free whole rice flour from Salud e Imaginación (Barcelona, Spain). Instant yeast was provided by Lesaffre (Marcq-en-Baroeul, France). Choline chloride was purchased from Sigma Aldrich (St. Louis, USA). Xanthan gum was provided by Grupo Desarrollo (Valencia, Spain). Sugar and salt were obtained in the local market.

Chemical composition of the flours was analyzed using standard ISO methods: moisture (712:2009); ash (2171:2007); protein (6634-2:2016); total fat (1185:2015) and the total carbohydrate content was estimated by difference. Wheat flour contained 14.93 g/100 g moisture, 0.9 g/100 g ash, 10.4 g/100 g protein, 1.6 g/100 g fat, and 72.17 g/100 g carbohydrates. In comparison, whole rice flour contained 12.96 g/100 g moisture, 1.14 g/100 g ash, 8.52 g/100 g protein, 2.28 g/100 g fat, and 83.72 g/100 g carbohydrates.

**Breadmaking process**

Three wheat containing flatbread recipes (coded G for gluten) and three gluten-free (GF) flatbreads recipes with varying levels of salt were developed. Those three recipes for each type of bread, were namely control (C), salt-reduced (Red), and choline chloride (CC). The hydration level to reach dough consistency (1.1 Nm) for each formulation was determined using Mixolab (Chopin, Paris, France). In the case of wheat containing recipes, the control recipe (G-C) consisted of 100 g of wheat flour, 58.7 g/100 g water, 1 g/100 g dry yeast, and 1.5 g/100 g salt. The salt reduced recipe (G-Red) was formulated with a 50% reduction in salt content, while the choline chloride recipe (G-CC) had a 50% reduction in salt combined with the addition of 0.375 g/100 g choline chloride. This substitution level was based on the findings presented by Le-Bail et al. at the CIGR Section VII Technical Symposium (Guangzhou, China, 2013). In the case of gluten-free formulations, the control recipe (GF-C) consisted of 100 g of rice flour, 59.9 g/100 g water, 1 g/100 g dry yeast, 1.5 g/100 g xanthan gum, 2 g/100 g sugar and 1.5 g/100 g salt. Like in the case of the gluten containing recipes, salt reduced gluten-free recipe (GF-Red) was formulated with a 50% reduction in salt content, while the choline chloride recipe (GF-CC) had a 50% reduction in salt combined with the addition of 0.375 g/100 g choline chloride.

Ingredients were mixed in two steps. First water was mixed using a Robot Coupe RM8 (Robot Coupe, Vincenne, France) with all the solids, with the exception of salt/CC, for 3 minutes at low speed. Then, after adding salt/choline chloride, kneading was followed by fast mixing for 5 minutes. The dough was covered with cloth, rested for 15 min (25ºC, RH = 50%), and divided into portions of 70 g. The proofing was for 40 min (25ºC, RH = 50%), and the portions were manually laminated to 2 mm of thickness. Finally, sheeted doughs were baked at 300ºC for 2 min (Bauuman Tech, S.L., Valencia, Spain) in a deck oven and cooled down at room temperature for 30 min before packing in sealed plastic pouches. Two batches were prepared for each type of bread.

**Dough characteristics**

Moisture content, proofing capacity and hardness of the dough were determined. Dough expansion was examined by placing 50 g dough into a graduated cylinder and the volume increase was recorded after 40 minutes of proofing. The result was expressed as an increment of dough volume (mL). The dough moisture was analyzed in two steps using standard method (ISO 712:2009). The texture of the dough was determined in a Texture Analyzer TA-XT2i (Stable Micro Systems, Surrey, UK) according to Gasparre et al., [16]. Dough pieces (20 g) were compressed up to 70% at a speed of 1 mm/s with an activation force of 5 g, using the compression probe P/75 and a 5 kg load cell. Dough hardness (g) was the recorded parameter. Six replicates were performed for each type of bread.

**Flatbread technological properties**

Weight loss, moisture content, tensile force, and color were determined in the flatbreads (FB). The baking weight loss (%) was calculated by the difference between the weight of flatbread dough before and after baking. The moisture of the flatbread was determined in two steps according to the standard method (ISO 712:2009). The Texture Analyzer TA-XT plus (Stable Micro Systems, UK) equipped with a 5 kg load cell was used to measure the tensile texture. The flatbread was cut into 2 mm wide strips, and the tensile grips were used. The parameters obtained were the strength (N) and the extensibility (mm). The color was analyzed on the top and bottom (burnt side included) with a colorimeter CR-400, Konica Minolta (New Jersey, USA) after standardization with a white calibration plate, using the CIE-*L*a*b** system. The flatbread images were captured using a scanner (Epson V550, Suwa, Japan). Six replicates were performed for type of bread.

**Starch *in vitro* digestion**

The flatbreads were subjected to enzymatic hydrolysis with porcine pancreatic α-amylase (0.9 U/mL) incubated for 3 hours at 37ºC, as previously reported [15]. The resistant starch was solubilized with NaoH (1.7 M) and incubated with amyloglucosidase (143 U/mL) for 30 min at 50ºC. The aliquots were analyzed using glucose oxidase–peroxidase (GOPOD) provided by Megazyme (Megazyme International Ireland Ltd., Bray, Ireland) to obtain the glucose content The parameters recorded were rapidly digestible starch (RDS), starch fraction hydrolyzed in the first 20 min; slowly digestible starch (SDS), starch fraction hydrolyzed from 20 to 120 min, total digestible starch (DS) and resistant starch (RS), as the amount of hydrolyzed and unhydrolyzed starch after 24 hours of incubation, respectively. The experimental data was modelized using the nonlinear Exponential model of Box-Lucas **Eq. (1)** to obtain *k* (kinetic constant) and C_∞_ (maximum hydrolysis). The area under the curve between 0-180 min (AUC) was calculated. Four replicates were performed for each blend.

$C=C_{\infty}(1-e^{-kt})$ **Eq. (1)**

**Statistical analysis**

Analysis of variance (ANOVA) was performed using OriginPro, Version 2022b. (OriginLab Corporation, Northampton, MA, USA). Fischer test with 95% of confidence level was to estimate significant differences among experimental mean (*p* < 0.05). The results were expressed as mean values ± standard deviation and the statistical comparisons were performed separately for gluten-containing and gluten-free flatbreads. Principal Component Analysis (PCA) was performed to identify differences among samples, considering salt reduction and the presence or absence of gluten in flatbreads.
